# ZnO QDs/GO/g-C_3_N_4_ Preparation and Photocatalytic Properties of Composites

**DOI:** 10.3390/mi14081501

**Published:** 2023-07-26

**Authors:** Zhixin Ren, Huachao Ma, Jianxin Geng, Cuijuan Liu, Chaoyu Song, Yuguang Lv

**Affiliations:** 1 College of Pharmacy, Jiamusi University, Jiamusi 154000, China; 2 School of Chemistry and Chemical Engineering, Shanghai Jiao Tong University, Shanghai 200240, China

**Keywords:** zinc oxide, graphene oxide, graphite phase carbon nitride, composites, photocatalysis, degradation

## Abstract

Using an ultrasound-assisted chemical technique, ZnO quantum dot and ZnO composites were created. The optical characteristics and structural details of these composites were examined using TEM, XRD, XPS, FT-IR, UV-vis, and BET. The results revealed that both the ZnO quantum dot composite and ZnO composite exhibited outstanding optical properties, making them suitable for photocatalytic reactions. In order to analyze the photocatalytic performance, a degradation experiment was conducted using Rhodamine B solution as the simulation dye wastewater. The experiment demonstrated that the degradation of Rhodamine B followed the first-order reaction kinetics equation when combined with the photocatalytic reaction kinetics. Moreover, through cyclic stability testing, it was determined that the ZnO QDs-GO-g-C_3_N_4_ composite sample showed good stability and could be reused. The degradation rates of Rhodamine B solution using ZnO-GO-g-C_3_N_4_ and ZnO QDs-GO-g-C_3_N_4_ reached 95.25% and 97.16%, respectively. Furthermore, free-radical-trapping experiments confirmed that ·O^2−^ was the main active species in the catalytic system and its photocatalytic mechanism was elucidated. The photocatalytic oxidation of ZnO quantum dots in this study has important reference value and provides a new idea for the subsequent research.

## 1. Introduction

At present, the living environment of human beings is deteriorating [1,2,3,4]. Our excessive dependence on fossil fuels leads to serious energy crises, ecological damage and environmental pollution, and other problems which have attracted the continuous study of scientific researchers. A large amount of polluted water flows into rivers, lakes, and the sea, causing serious pollution of water sources and the death of a large number of marine lives. In order to solve these problems, researchers have conducted a large number of exploratory experiments [5], looking for ways to solve water pollution and new, environmentally friendly, and economical energy that can replace non-renewable energy [6,7]. Therefore, photocatalytic technology has become an effective and sustainable technology [8].

In recent years, researchers from various disciplines have extensively studied zinc oxide (ZnO) due to its excellent electrical and optical properties [9]. QDs, on the other hand, are quasi-zero-dimensional nanomaterials that exhibit the quantum confinement effect in all three spatial dimensions (XYZ), resulting in the confinement of charge carriers in three dimensions [10,11]. Compared to bulk materials, ZnO QDs possess exceptional physical, chemical, and interfacial properties [12,13,14,15]. As a result, they exhibit distinct characteristics, making them promising candidates for various applications such as solar cells [16], catalysis [17], photoelectric devices [18], high-efficiency energy storage electrodes [19], ultraviolet photodetectors [20,21], anticancer drug carriers [22], and light-emitting electrochemical cells [23]. To achieve high-purity ZnO QDs with good crystal form, soft chemical methods like the sol–gel method [24], hydrothermal method [25], and microemulsion method [26] are commonly employed for their synthesis.

g-C_3_N_4_, a representative nonmetallic semiconductor, has a highly delocalized π-conjugated system formed by the hybridization of CN atoms with sp^2^ [27]. Despite its recent emergence in the public eye, g-C_3_N_4_ has gained attention due to its excellent chemical and thermal stability, affordability, availability, and environmental characteristics [28,29,30]. It has been identified as one of the most promising photocatalysts in the realm of wastewater treatment [31]. To enhance the catalytic activity and inhibit the recombination of photogenerated charge, g-C_3_N_4_ is often utilized as a substrate to support other semiconductors [32,33,34].

Graphene oxide (GO), a carbon-based nanomaterial widely used for adsorption and catalytic degradation, is an ideal material for improving the performance of wide bandgap semiconductor photocatalysts due to its high specific surface area and tunable structure [35]. It is also a promising nanomaterial widely used for energy storage [36], electronic sensors [37], and antimicrobial [38]. Graphene has special properties such as high electron mobility [39], good electrical conductivity [40], optical transgressivity [41], and good chemical stability [42], which gives it excellent performance in adsorption properties and photocatalytic activity. Additionally, the defects in GO’s own structure can be utilized as active centers to enhance its photocatalytic activity [43]. A self-powered potential humidity conversion technique, for instance, was developed by Lei et al. [44] in 2021. It controls the observed potential difference between two electrodes by humidifying a GO solid electrolyte. In non-contact sensing applications, it has demonstrated outstanding performance. Using bioreductive graphene oxide derived from organisms in 2023, Zhou’s group demonstrated excellent antibacterial efficacy [45]. A straightforward and adaptable technique to increase the electrical conductivity of macroscopic graphene materials in a controlled manner was put out by Chen et al. in 2023 [46]. After refining the process for making graphene in 2017, Chen’s team [47] discovered that it had outstanding dispersion and stability in organic solvents.

Photocatalysis is an advanced technology that utilizes solar energy to drive chemical reactions that are difficult or impossible to achieve in the absence of light. It has the potential to degrade hazardous organic pollutants in wastewater, thus improving the environment. In recent studies, researchers have explored various materials and composites to enhance the photocatalytic performance. For instance, Chen’s group combined g-C_3_N_4_ with carboxylated graphene oxide (GO-COOH) after acid treatment, resulting in a 5.42 times higher efficiency for photocatalytic degradation compared to g-C_3_N_4_ alone [48]. Jung’s group synthesized g-C_3_N_4_/ZnO (CNZ) composites with different morphological structures and found that the structure strongly affects the photocatalytic performance during the degradation of methylene blue [49]. Li’s group prepared GO/g-C_3_N_4_ complexes using graphene oxide and graphitic carbon nitride to achieve efficient photocatalytic hydrogen production. The goal was to utilize inexpensive carbon-based materials to achieve efficient collection and transport of photogenerated carriers during the water photolysis process [50]. Similarly, KRavichandran’s group prepared ZnO:Ag/GO nanocomposites for the degradation of the dye methylene blue using an automatic jet atomizer spray pyrolysis technique. Through various characterizations, they successfully demonstrated the preparation of the complexes and proposed a possible mechanism for the degradation process [51].

In this paper, composite photocatalysts of GO, g-C_3_N_4_ doping amount of ZnO-GO-g-C_3_N_4_, and ZnO QDs-GO-g-C_3_N_4_ were synthesized using an ultrasound-assisted chemical method. The synthesized ZnO QDs complexes were characterized using modern techniques such as TEM, XRD, XPS, FT-IR, and UV-vis for structural and optical analysis.

## 2. Materials and Methods

### 2.1. Reagents and Instruments

Silane coupling agent was purchased from Aladdin Co., Ltd. (Beijing, China), Rhodamine B(RhB) was purchased from Aladdin Industries (Beijing, China), Ammonium oxalate, Tert-butanol, P-benzoquinone was purchased from Shanghai Jining Industrial Co., Ltd. (Shanghai, China), and anhydrous ethanol was purchased from Tianjin Kaitong Chemical Reagent Co., Ltd (Tianjin, China).

Ultraviolet Spectrophotometer (UV-2550) was purchased from Shimadzu Company Tokyo, Japan; X-ray diffractometer (3DMAX-IIIC) was purchased from Nippon Shinosu Co., Ltd.; and Fourier Transform Infrared Spectrometer (NEXUS-670) was purchased from Denver, CO, USA.

### 2.2. Preparation Method of ZnO-GO-g-C_3_N_4_

0.1 g-C_3_N_4_, 1 g ZnO and 0.07 g graphene oxide were weighed separately and dispersed in 50 mL anhydrous ethanol and ultrasound for 1 h. The sonicated graphene oxide suspension was added to a flask and the temperature was raised to 120 °C. The ZnO suspension was added and mechanically stirred for 2 h. The g-C_3_N_4_ sonicated solution was added to the graphene oxide flask in a hydrothermal reactor at 140 °C. The solid product obtained was washed by alcohol and dispersed into 10 mL of anhydrous ethanol. Nano-ZnO-GO-g-C_3_N_4_ was dried under vacuum in a vacuum drying oven for 24 h. The product was labeled as ZCG.

### 2.3. Preparation of Water-Soluble ZnO QDs-GO-g-C_3_N_4_

To prepare the ZnO QDs powder, 1.1 g of (CH_3_COO)_2_Zn-2H_2_O was stirred with 50 mL of anhydrous ethanol. The mixture was then condensed and refluxed at 85 °C for 2 h. After cooling it in an ice bath to 0 °C, this solution was labeled as solution 1. A separate solution, labeled as solution 2, was prepared by stirring 0.392 g of KOH with 5 mL of anhydrous ethanol. Solution 2 was added dropwise to solution 1 under sonication. After bringing the mixture to room temperature, 1.5 mL of H_2_O and 800 µL of APTES were added dropwise and stirred. The resulting solution was then centrifuged at 6000 r/min for 10 min. The supernatant was removed, and the precipitates were washed three times with alcohol. The washed precipitates were then placed in a blast drying oven at 60 °C for 2 h to obtain water-soluble ZnO QDs powder. The photocatalytic activity and cyclic stability of the catalyst composite samples were analyzed by performing visible light degradation of RhB. Furthermore, the active species and photocatalytic mechanism of the composite samples were elucidated in this analysis.

0.1 g-C_3_N_4_, 1 g ZnO QDs, and 0.07 g GO were weighed and dispersed in 50 mL anhydrous ethanol and then ultrasonicated for 1 h. In a flask, the GO suspension was sonicated and heated to 120 °C. The ZnO QDs suspension was slowly added drop by drop to the GO suspension and mechanically stirred for 2 h. Simultaneously, a solution of g-C_3_N_4_ was sonicated at 140 °C and added to the GO bottles in the hydrothermal reactor. The resulting solid product was washed with alcohol and then dispersed in 10 mL absolute ethanol. Nano-ZnO-GO-g-C_3_N_4_, labeled as ZCG QDs, was obtained by vacuum drying the product for 24 h at 80 °C in a vacuum dry box.

A suitable amount of silane coupling agent (APTES) can be added during the synthesis process to cover the ZnO QDs, forming a SiO_2_ shell layer on their outer surface. This process results in the production of ZnO/SiO_2_ quantum dots that exhibit improved stability and performance [52,53]. The addition of the SiO_2_ shell layer serves multiple purposes. Firstly, it prevents the quantum dots from coming into contact with other substances, thus enhancing their protection and stability. Secondly, it acts as a barrier against potential damage from various factors. Additionally, the growth of an amorphous SiO_2_ shell layer on the surface of ZnO increases the distance between the particles of ZnO QDs, leading to improved dispersion [54].

### 2.4. Degradation of Rhodamine B Catalyzed by Visible Light

The catalyst prepared by weighing a specific mass was dispersed in 50 mL Rhodamine B (10 mg/L) solution, while avoiding exposure to light. The dark reaction was then conducted and stirred for 60 min in order to achieve the adsorption–desorption equilibrium. Following this, a 5 mL solution was extracted and subjected to centrifugation to remove any impurities. The clear upper portion of the extraction was left undisturbed overnight and subsequently placed in a UV-vis absorption spectrometer for sample testing. Subsequently, under visible light reaction condition, the mixture was stirred for 360 min. At intervals of 60 min, a 5 mL solution was drawn using a pipette for sample analysis. Similar to the previous step, the extracted solution was centrifuged, the upper portion cleared overnight, and then analyzed in a UV-vis absorption spectrometer to determine the absorption of Rhodamine B at each specific time point.

## 3. Results and Discussion

### 3.1. TEM and SEM

**Figure 1:**
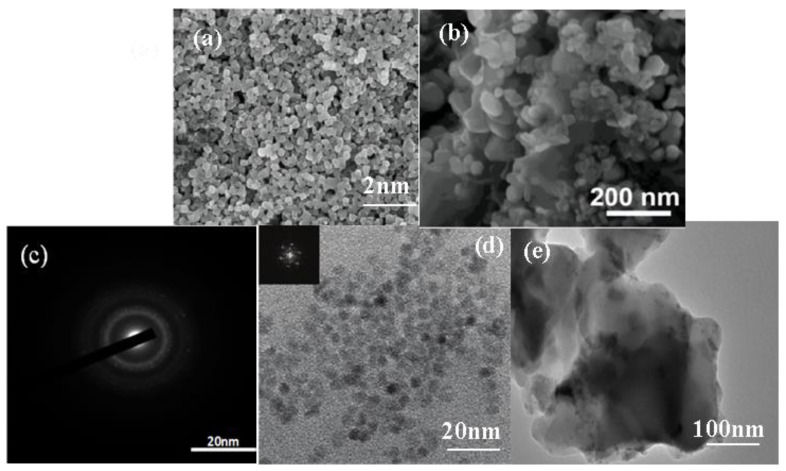
(**a**) FESEM image of ZnO nanoparticles (**b**) FESEM image of ZnO-g-C_3_N_4_-GO. (**c**,**d**) Transmission electron microscopy image of ZnO QDs (**e**) TEM image of ZnO QDs-GO-g-C_3_N_4_ nanoparticles.

**Figure 2:**
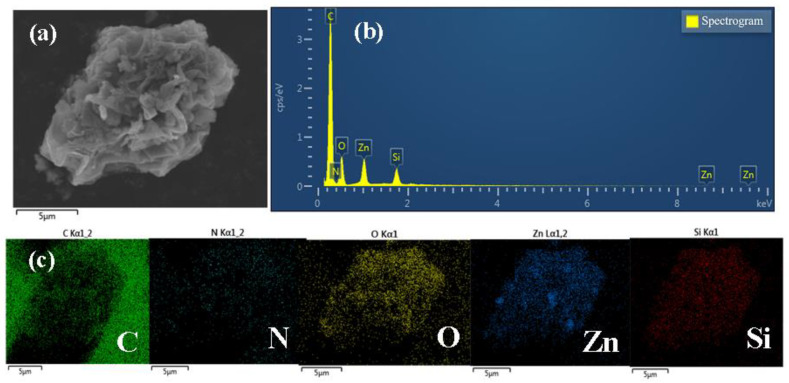
(**a**) SEM, (**b**) EDS, and (**c**) elemental mapping images of ZCG QDs.

According to Figure 1a, it can be intuitively found that the ZnO nanoparticles were composed of small crystals with good crystallization, and the morphology shows a relatively regular particle structure with pores between the nanoparticles, and the size of all the particles was relatively uniform and the surface was smooth. Figure 1b shows that the surface morphology of ZCG was also small nanoparticles, and the distribution of particles was looser, and an obvious lamellar structure appeared, with nanoparticles closely loaded on the lamellar structure. And the addition of g-C_3_N_4_, GO could inhibit nanoparticle agglomeration, which may be due to the fact that g-C_3_N_4_, GO contains many oxygen-containing functional groups on it, providing a connection point for its tight connection with ZCG [55]. Figure 1c shows the electron diffraction pattern of ZnO QDs. The electron diffraction pattern of ZnO QDs had a broadened circular spot and low brightness, which appears as “amorphous” phenomenon. This is probably because the electron waves could not completely pass through the amorphous shell layer on the surface of ZnO, resulting in scattering back to the amorphous siloxane shell layer on the surface [56]. This result is consistent with the reduced intensity of the characteristic peaks of the core-shell quantum dots in the following XRD characterization results. Figure 1d shows that an envelope layer was formed around the ZnO QDs due to the addition of silane coupling agent (APTES) during the preparation of ZnO QDs. It could better isolate the quantum dots from each other and enhance their monodispersity, so the ZnO QDs were well dispersed [57]. The dark colored part with lattice display was the ZnO QDs core, and the surrounding light colored and darker than the background part was the amorphous SiO_2_ shell layer. It can be seen that the ZnO core and shell layer were not a simple mixture, and the silicone shell layer was uniformly wrapped around the ZnO QDs to form a core-shell structure [58]. Figure 1e shows the TEM morphology of the ZCG QDs ternary composites, from which it can be seen that an obvious lamellar structure appeared, and the nanoparticles were tightly loaded on the lamellar structure. The dispersion of ZCG QDs complexes was significantly better than that of ZnO QDs, indicating that the addition of g-C_3_N_4_. GO could inhibit the aggregation of nanoparticles. This may be due to the presence of many oxygen-containing functional groups on g-C_3_N_4_, GO, which provides attachment points for their tight junctions. The individual elements can be clearly seen according to Figure 2.

### 3.2. XRD

**Figure 3:**
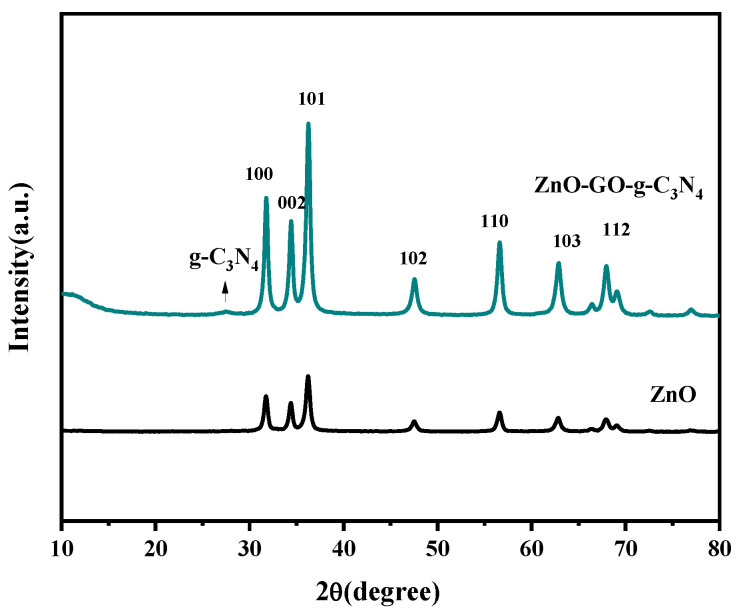
XRD plots of the ZnO and ZnO-GO-g-C_3_N_4_ complexes.

Figure 3 shows the XRD pattern of the ZnO samples, in which seven distinct characteristic diffraction peaks appeared at 2θ of 31.75°, 34.44°, 36.25°, 47.54°, 56.55°, 62.87°, and 67.92°, attributed to seven crystal planes ZnO (100), (002), (101), (102), (110), (103), and (112), respectively (JCPDs No. 36-1451) [59]. The product can be determined as ZnO of fiber zinc ore structure. As can be seen from the graph, the peaks are clearly located and shaped. There were no miscellaneous peaks, indicating that the sample nano-ZnO had the best crystallinity. The characteristic diffraction peaks of each component appeared in the ZCG ternary complex. The characteristic diffraction peaks were consistent with the ZnO standard card, and no other impurity phases appeared. The outcomes demonstrated that there was no change in the structural characteristics of each component during the composite process. The produced ZCG had lattice constants of a, b, and c = 3.24 °A and 5.19 °A, respectively. The outcomes were consistent with other works in the literature [60]. According to the Scherrer formula, the average crystallite size of the produced ZnO nanoparticles could be calculated. The equation D = kλ/β cos θ, where B is the crystalline size, expressed in nm, and is an X-ray wavelength (1.5406 °A), full width at half maximum angle for the XRD at half maximum intensity highest peak. The results showed that the average grain size of the measured ZCG was 31.37 nm [61].

**Figure 4:**
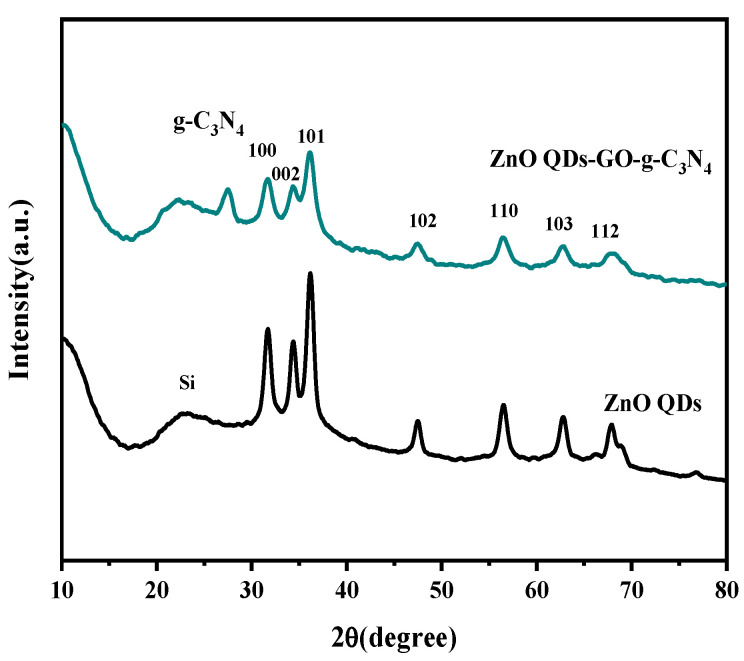
XRD plots of the ZnO QDs and ZnO QDs-GO-g-C_3_N_4_ complexes.

As can be seen from Figure 4, seven peaks appeared in seven crystal planes, namely 31.769° on (100) plane, 34.421° on (002) plane, 36.252° on (101) plane, 47.538° on (102) plane, and 56.602° on (110) plane, respectively. (103) side 62.862°, (112) side 67.961°. It can be seen that the diffraction peaks and crystal surfaces in the XRD images of ZnO QDs corresponded to those in the standard card (JCPDs 36-1451), without changing the crystal structure of ZnO itself. The hexagonal wurtzite structure of ZnO QDs could be obtained for ZCG QDs. The addition of GO and g-C_3_N_4_ did not damage the crystal form of ZCG QDs. Compared with ZnO QDs, ZCG QDs ternary composite had wider peak shape, smaller particle size, and more active photocatalysis. The Scherrer equation, D = kλ/β cos θ, calculates the average crystal (diameter), where k is the structural constant, k = 0.9, d is the size of the ZCG QDs, is full width at half maximum, and is the Bragg angle [62]. For the calculation of the size of ZCG QDs, we used the signal of the (102) with 2θ = 47.65°, and the diameter was found to be equal to 6.32 nm.

### 3.3. XPS

**Figure 5:**
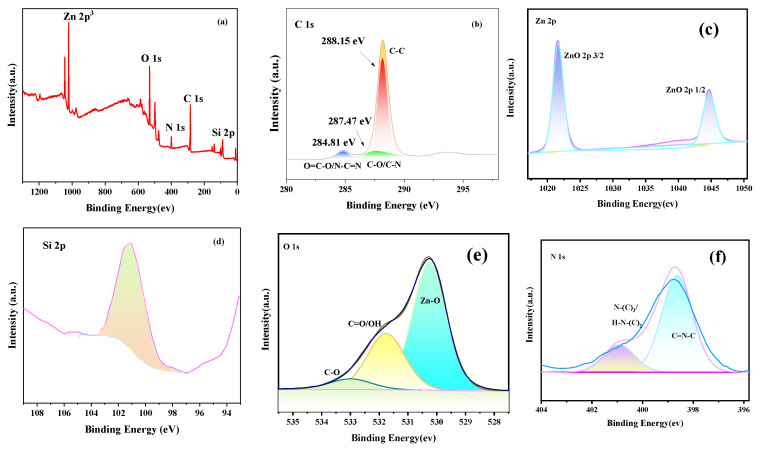
Full-spectrum analysis diagram of the ZCG QDs composite samples and the XPS diagram of the four elements. (**a**) total spectrum; (**b**) C spectrum; (**c**) Zn spectrum; (**d**) Si spectrum; (**e**) O spectrum; (**f**) N spectrum.

The whole XPS spectra of the ZCG QDs composite material is shown in Figure 5a, and the peaks of four elements C, N, O, and Zn were detected, and there were no other miscellaneous peaks in the figure. In Figure 5b, there are three characteristic peaks with binding energy of 284.8, 286.4, and 288.7 eV corresponding to oxygen-containing active groups such as C-C and sp^3^ hybridized C atom (C-(N)_3_), conjugate (C=O), and carboxyl group (O=C-OH), respectively [63]. In Figure 5c, the energy difference between the orbital spin splitting peaks (2p^3/2^ and 2p^1/2^) at 1022.4 eV and 1045.6 eV is about 23 eV, which is the typical binding energy of Zn^2+^ [64]. Also, Figure 5d shows the Si 2p core-level spectrum with a main peak at 101.54 eV (100%) and is assigned to Si atom bounded to oxygen and carbon (C–Si–O) in the APTES molecule. [65,66] These results confirm the success of grafting or capping of APTES with ZnO QDs. In Figure 5e, three characteristic peaks of O 1s are Zn-O, C=O/-OH and C-O, and the binding energies are located at 530.3, 531.0, and 532.2 eV, respectively. The peaks with a binding energy of 531.0 eV and 532.2 eV correspond to the OH^−^ and O^2−^ of ZnO adsorbed on the material surface, respectively [67]. Figure 5f the 398.5 eV and 399.8 eV peaks in N 1s orbit correspond to sp^2^ aromatic group N (C=N-C), and tertiary N (N-(C)_3_/H-N-(C)_2_), respectively [68]. A third peak with high binding energy at 400.9 eV was attributed to quaternary N in the aromatic ring. Through element type analysis, ZnO QDs, GO, and g-C_3_N_4_ can be well coupled, and this will aid in enhancing the composite’s catalytic activity for visible light.

### 3.4. FT-IR

**Figure 6:**
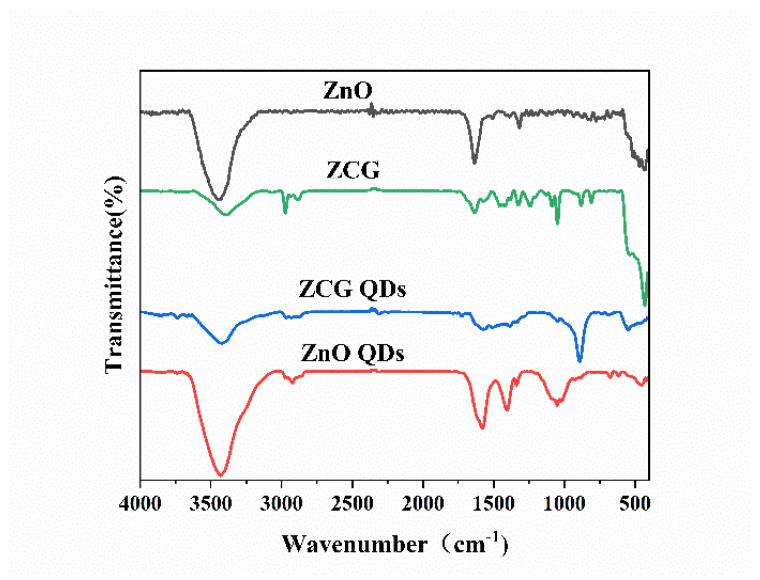
FT-IR plots of ZnO-GO-g-C_3_N_4_, ZnO QDs-GO-g-C_3_N_4_ samples.

As seen in Figure 6, the absorption peak at 459 cm^−1^ was from the Zn-O bond of ZnO, and the absorption peaks around 1571 cm^−1^ and 1400 cm^−1^ could be attributed to the asymmetric and symmetric C=O stretching vibrations of Zn(OAc)_2_. Furthermore, on pure phase ZnO, the broad peaks around 3404 cm^−1^ corresponded to the -OH groups on the surface of ZnO QDs. For NH_2_-ZnO QDs, the absorption peaks in the region from 3000 to 3500 cm^−1^ could be attributed to the peak overlap of the NH_2_ group of APTES with the -OH group of ZnO QDs. In addition, the absorption peak at 1002 cm^−1^ could be attributed to the stretching vibration of the Si-O band in APTES. The comparison shows that APTES had been successfully used as surface modification and modified to the surface of ZnO QDs. The ZnO-GO-g-C_3_N_4_ and ZnO QDs-g-GO-g-C_3_N_4_ composites have typical shrinking vibration peaks near 500 cm^−1^ for ZnO and also typical NH_2_-ZnO quantum dot absorption peaks that overlap with the -OH group peaks of ZnO quantum dots in the 3000~3500 cm^−1^ region. Moreover, the typical stretching vibration absorption peaks of g-C_3_N_4_ in the vicinity of 1240~1640 cm^−1^ and 810 cm^−1^ were found. GO material at 1089 cm^−1^ and 1623 cm^−1^ corresponded to C-O and C=C stretching vibration absorption peaks, respectively. The results show that ZnO and ZnO QDs were successfully combined with GO and g-C_3_N_4_.

### 3.5. DRS Analysis

**Figure 7:**
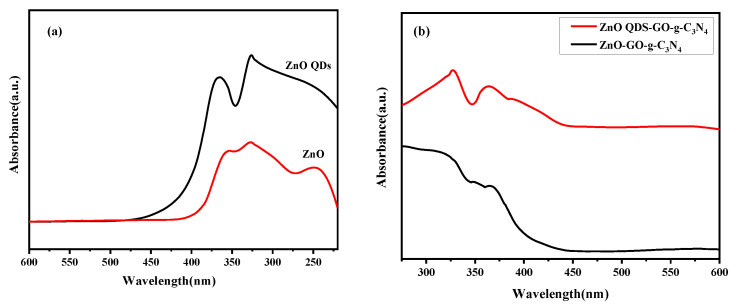
(**a**) UV-visible diffuse reflectance of ZnO and ZnO QDs; (**b**) UV-visible diffuse reflectance of ZCG and ZCG QDs.

It can be seen from Figure 7 that compared with ZCG, the surface modification of the compound ZCG QDs reduced the non-composite radiation center and enables the material to have stronger exciton absorption. However, there was no significant change in the position of the exciton absorption peak and half-peak width, indicating that the main carrier of optical absorption reaction was ZnO core. In terms of the absorption range position, there was a small amount of redshift phenomenon. The reason for this phenomenon was that the radius of the material is increased slightly under the action of the reaction conditions. ZCG QDs nanocomposite had better photocatalytic performance.

### 3.6. BET Analysis

**Figure 8:**
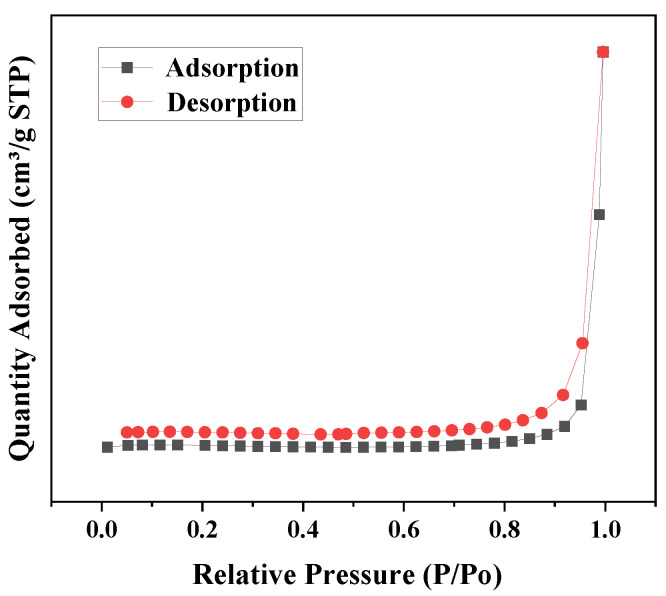
Nitrogen adsorption diagram of ZnO QDs-GO-g-C_3_N_4_ nanocomposites.

To further investigate the microstructure of the prepared composites, the specific surface area and pore size of the composites can be measured and determined using nitrogen adsorption–desorption experiments. As shown in Figure 8, the ZCG QDs nanocomposite catalyst has a type IV adsorption–desorption isothermal curve and hysteresis phenomnon, which can prove that the photocatalyst has a mesoporous structure, where the hysteresis phenomenon is related to the shape and size of the pores. The pore-like structure may be generated due to the overloading of ZnO QDs covering the g-C_3_N_4_ surface. The fomation of pore channels facilitates the adsorption of organic molecules to the active centers on the surface of the catalytic material and can enhance the light-absorbing properties of the material [69]. It is shown that the material has a high specific surface area and porous structure, which can significantly reduce the transmission distance of photogenerated eletron-hole to the photocatalytic material and effectively inhibit the recombination of photogenerated electron-hole [70]. The surface area of ZCG QDs was 1.3999 m^2^ g^−1^, and the average pore size and pore volume calculated by the Barrett–Joyner–Hallenda (BJH) method were 19.5025 nm and 0.014444 cm^3^ g^−1^, respectively.

### 3.7. Photocatalytic Performance

The performance of the photocatalysts of ZCG QDs ternary composites was investigated. The experimental conditions were visible light, 50 mL, 10 mg·L^−1^ of Rhodamine B solution was prepared, and 0.02 g of ZCG QDs ternary compound was weighed. pH of the solution was adjusted to pH = 7, and the dark reaction test was completed at room temperature with the absorption time set to 1 h, and then the light reaction test was completed with the absorption time set to 6 h.

**Figure 9:**
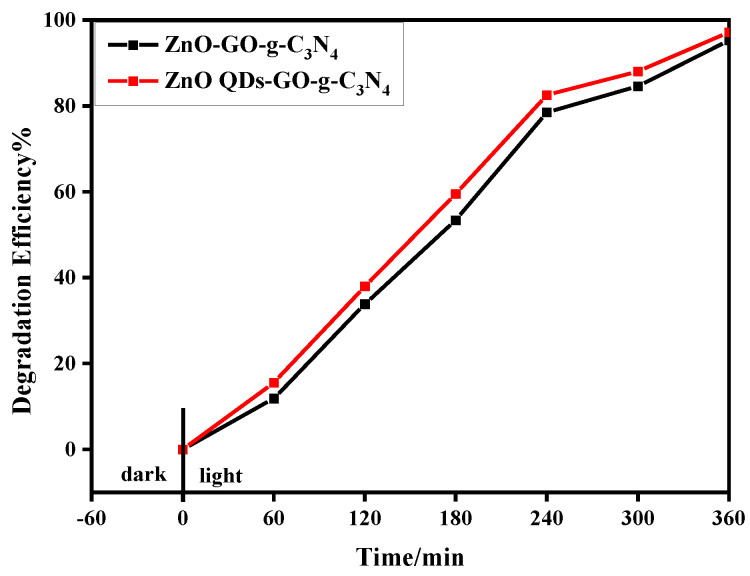
Photocatalytic degradation curves of ZCG, ZCG QDs ternary composites.

It can be seen from Figure 9 that after 360 min of visible light irradiation, the degradation rates of the RhB solution by ZCG and ZCG QDs were 95.25% and 97.16%, respectively. This result is consistent with the previous structure and morphology analysis. Compared with ZCG, ZCG QDs composite had a smaller particle size, which raised the contact area for visible light absorption and boosts photocatalytic effectiveness. The ZCG QDs complex exhibited the highest photocatalytic efficiency.

### 3.8. Kinetic Study

**Figure 10:**
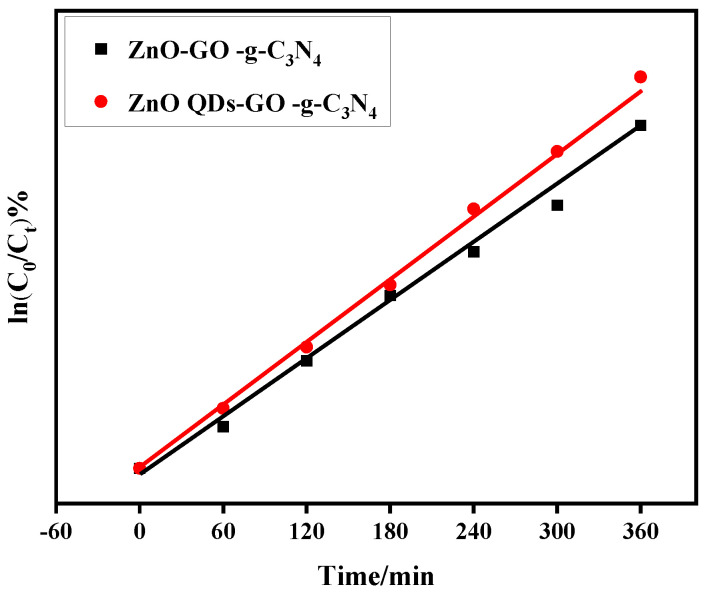
Primary reaction constants for photocatalytic degradation of RhB in ZCG, ZCG QDs composite samples.

To reach the equilibrium between adsorption and desorption, the composite sample underwent a dark reaction in RhB for 60 min. The start time of the light reaction and the conclusion of the dark reaction were both set at C_0_. The analysis of the relationship between ln(C_0_/C_t_) and time t revealed a linear relationship between ln(C_0_/C_t_) of each sample and the photocatalytic reaction time t. This relationship is depicted in Figure 10.

As can be seen from Table 1, ZCG QDs have a high k value, k = 0.00784 min^−1^, and a high RhB degradation rate. The correlation coefficient R^2^ of each ZCG QDs composite sample is greater than 0.9, which indicates that the samples conform to the D_RhB_ first-order reaction kinetic equation.

### 3.9. Cycling Stability

**Figure 11:**
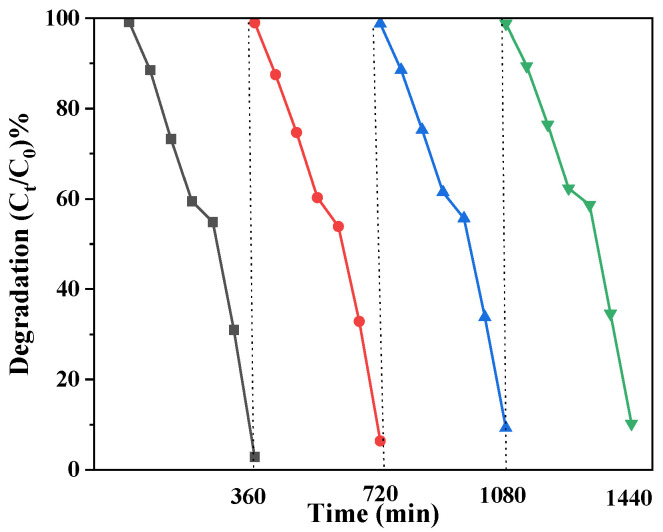
ZnO QDs-GO-g-C_3_N_4_ pHotocatalytic degradation of RhB stability.

By performing repeated photocatalytic degradation of RhB studies, we assessed the catalyst’s stability and reusability. The illumination time was controlled for 360 min. After filtration, ultrasonic, and drying, the samples were recycled. The photocatalytic deterioration of the catalyst sample after four circulation of testing is depicted in Figure 11 and Table 2.

According to Figure 9, the photocatalytic degradation rate decreased slightly after four cycles of experiments, which may be due to the occurrence of photocorrosion. Generally speaking, it still has good stable performance of repeated use.

### 3.10. Free Radical Capture

The photocatalyst was prepared by composite modification of ZnO QDs and semiconductor materials. It was applied to the degradation of polluted wastewater. The main active species in the degradation process of most semiconductor materials are ·O^2−^, ·OH, and h^+^. Ammonium oxalate (AO), while tert-butanol (TBA) and p-benzoquinone (BQ) were added into the quantum dot composite samples as trapping agents of superoxide radical (·O^2−^), hydroxyl radical (·OH) and hole (h^+^) to determine the main active species of photocatalytic degradation of RhB. 

**Figure 12:**
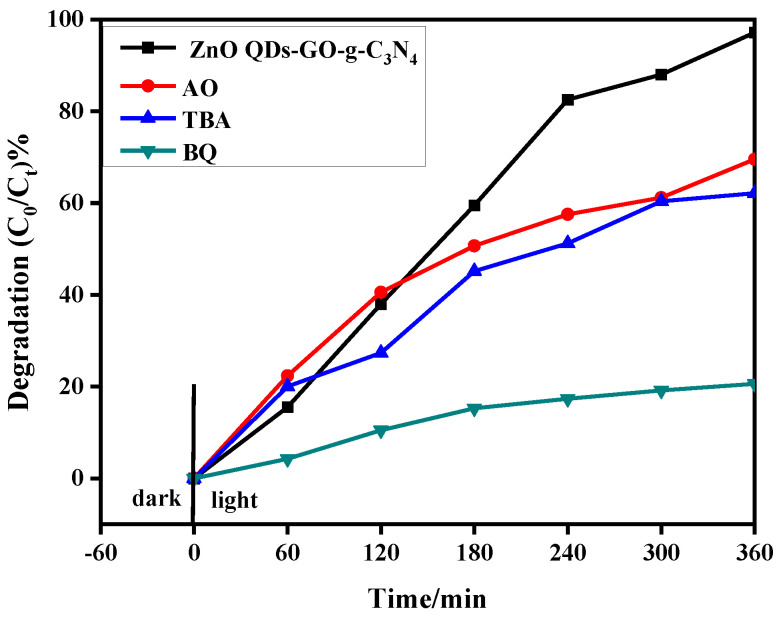
Photocatalytic performance of ZCG QDs on RhB after addition of AO, TBA, and BQ.

As shown in Figure 12, it can be found that the ZCG QDs sample D_RhB_ = 97.16%. The photocatalytic degradation of the materials could be greatly inhibited by the addition of three kinds of trapping agents. Therefore, in the visible ring, ·O^2−^, ·OH, and h^+^ were the main catalytic active components. After 360 min of light exposure, they reached 69.56%, 62.13%, and 20.16%, respectively, in which the superoxide radical (·O^2−^) played the most important role.

### 3.11. Photocatalytic Mechanism

**Figure 13:**
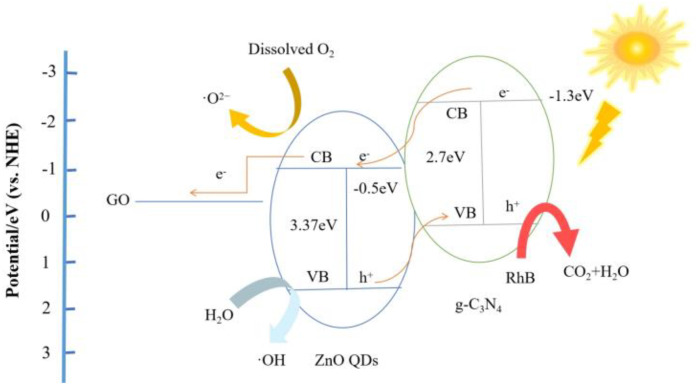
Schematic diagram of the pHotocatalytic reaction mechanism of ZGC QDs under visible light irradiation.

Compared to the standard hydrogen electrode (NHE), the Fermi energy level of GO, the conduction band edge (CBE) of ZnO, and the conduction band edge of g-C_3_N_4_ are −0.08 eV, −0.5 eV, and −1.3 eV, respectively. Therefore, under light, g-C_3_N_4_ absorbs light energy and transfers electrons from the valence band to the conduction band. Electrons from the conduction band of g-C_3_N_4_ leap to the conduction band of ZnO, and electrons from the valence band of ZnO are transferred to the valence band of g-C_3_N_4_, and the whole process transfers electrons and holes and prevents the compounding of photogenerated electrons and holes. After the addition of the third element material GO, electrons from the conduction band of ZnO are transferred to GO, in which GO acts as an acceptor for the electrons and effectively inhibits the compounding of photogenerated electrons and holes. The carriers then migrate to the composite surface and generate hydroxyl and superoxide radicals with water and dissolved oxygen, respectively, which in turn oxidize RhB. This is shown in Figure 13.

### 3.12. Comparative Analysis

We reviewed the relevant information and compared the degradation of similar substances, as shown in Table 3, and we believe that our article has a relatively high degradation rate.

## 4. Conclusions

In this paper, ZnO and ZnO QDs and their complexes were prepared. Using its photocatalysis in wastewater degradation, this study provides a theoretical basis for better solutions to the actual needs of human life. Graphene oxide, g-C_3_N_4_, and ZnO QDs samples were doped to prepare composite materials. The surface morphology, crystal structure, element types, and functional groups of the materials were measured by modern instruments. RhB degradation was used to gauge the produced materials’ photocatalytic performance, and the optimum photocatalytic reaction conditions and principles were analyzed and explored in combination with the degradation kinetics equation, cyclic stability test, and free radical capture test. It was found that the doping of GO and g-C_3_N_4_ changed the degradation pathway of RhB, which increased the adsorption performance, promoted the separation of photogenerated carriers, and increased the use efficiency of light. The D_RhB_ = 95.25% for the ZCG sample and 97.16% for the ZCG QDs sample were obtained. All of them conformed to the first-order reaction kinetic equation, had good stability, and could be recycled. According to the free radical trapping experiment, ·O^2−^ was the main active species of ZCG QDs samples.

## Figures and Tables

**Table 1:** Kinetic analysis of photocatalytic reactions of ZCG, ZCG QDs composite samples.

Sample Name	Regression Equation	*k*	*R* ^2^
ZnO-GO-g-C_3_N_4_	*y* = 0.4768*x* − 0.4780	0.00778	0.9822
ZnO QDs-GO-g-C_3_N_4_	*y* = 0.559*x* − 0.6134	0.00784	0.9826

**Table 2:** The catalyst’s photocatalytic degradation after four uses.

Number of Use	1	2	3	4
decolourization ratio (%)	97.16	93.64	90.76	89.78

**Table 3:** Comparison of similar degradation.

Articles	Degradation Rate
Our Articles	The degradation of RhB solutions by ZnO-GO-g-C_3_N_4_ and ZnO QDs-GO-g-C_3_N_4_ could reach 95.25% and 97.16%.
[71]	The photocatalytic properties of the nanoparticles were established by degrading methylene blue (MB) dye in response to solar radiation. The degradation rate was up to 88% over a period of 270 min.
[72]	Methylene blue degrades approximately 92% in 70 min.
[73]	The photocatalytic activity of the synthesized samples was studied for the degradation of peacock green (MG) dye under UV light irradiation. Among the studied samples, Zn_0.94_Ni_0.06_O was the better photocatalyst, degrading 77% of the dye under 4 h of irradiation.
[74]	Optimal photocatalyst loading of ZnO prepared by precipitation and sol-gel was required to reach 250 mg/L to achieve up to 81% and 92.5% dye degradation by ZnO.
[75]	The anionic dye (acid blue 113, AB113) was degraded by zinc oxide-termite hills complex (ZnO-TH) in aqueous solution and the maximum decolorization efficiency was found to be 92.21% under optimal conditions.
[76]	The degradation rate can reach 92%.
[77]	Synthesized nanoparticles show potential dye degradation efficiency under light irradiation (~90%)
[78]	ZnO/PUF showed good efficiency in the degradation of AB1 dyes, up to 86% and 65% at neutral pH, 4% H_2_O_2_, 240 min/sunlight and 75 min/UV irradiation time under UV and sunlight.
[79]	Biological ZnO NPs (10 mg/L) exhibited 91% degradation of methylene blue and 86% degradation of methyl orange.
[80]	The photocatalytic degradation of reactive yellow 186 (RY 186) dye was carried out under direct sunlight, and its degradation efficiency was 93.38%.

## Data Availability

Data not visible.

## References

[1] Rajapaksha P., Orrell-Trigg R., Shah D., Cheeseman K.B., Vu S.T., Ngo B.J., Murdoch N.R., Choudhury H., Yin D., Cozzolino Y.B. (2023). Chapman Broad spectrum antibacterial zinc oxide-reduced graphene oxide nanocomposite for water depollution. Mater. Today Chem..

[2] Vasantharaj S., Sathiyavimal S., Senthilkumar P., Kalpana V., Rajalakshmi G., Alsehli M., Elfasakhany A., Pugazhendhi A. (2021). Enhanced photocatalytic degradation of water pollutants using bio-green synthesis of zinc oxide nanoparticles (ZnO NPs). J. Environ. Chem. Eng..

[3] Ferdosi E., Bahiraei H., Ghanbari D. (2019). Investigation the photocatalytic activity of CoFe_2_O_4_/ZnO and CoFe_2_O_4_/ZnO/Ag nanocomposites for purification of dye pollutants. Sep. Purif. Technol..

[4] Yousefi H.R., Hashemi B. (2019). Photocatalytic properties of Ag@ Ag-doped ZnO core-shell nanocomposite. J. Photochem. Photobiol. A Chem..

[5] Reddy P.A.K., Reddy P.V.L., Kwon E., Kim K.-H., Akter T., Kalagara S. (2016). Recent advances in photocatalytic treatment of pollutants in aqueous media. Environ. Int..

[6] Mohamed W.A.A., Abd El-Gawad H.H., Mekkey S.D., Galal H.R., Labib A.A. (2021). Zinc oxide quantum dots: Confinement size, photophysical and tunning optical properties effect on photodecontamination of industrial organic pollutants. Opt. Mater..

[7] Banerjee A., Chattopadhyay S., Kundu A., Sharma R.K., Maiti P., Das S. (2019). Vertically aligned zinc oxide nanosheet for high-performance photocatalysis of water pollutants. Ceram. Int..

[8] Zhang S., Yang Y., Guo Y., Guo W., Wang M., Guo Y., Huo M. (2013). Preparation and enhanced visible-light photocatalytic activity of graphitic carbon nitride/bismuth niobate heterojunctions. J. Hazard. Mater..

[9] Ren S., Wang B., Zhang H., Ding P., Wang Q. (2015). Sandwiched ZnO@Au@Cu_2_O nanorod films as efficient visible-light-driven plasmonic photocatalysts. ACS Appl. Mater. Interfaces.

[10] Ye Y. (2018). Photoluminescence property adjustment of ZnO quantum dots synthesized via sol-gel method. J. Mater. Sci. Mater. Electron..

[11] Bajorowicz B., Kobylański M.P., Gołąbiewska A., Nadolna J., Zaleska-Medynska A., Malankowska A. (2018). Quantum dot-decorated semiconductor micro-and nanoparticles: A review of their synthesis, characterization and application in photocatalysis. Adv. Colloid Interface Sci..

[12] Jin Y.K., Voznyy O., Zhitomirsky D., Sargent E.H. (2013). 25th anniversary article: Colloidal quantum dot materials and devices: A quarter-century of advances. Adv. Mater..

[13] Yang W., Yang H., Ding W., Zhang B., Zhang L., Wang L., Yu M., Zhang Q. (2016). High quantum yield ZnO quantum dots synthesizing via an ultrasonication microreactor method. Ultrason. Sonochem..

[14] Laurenti M., Canavese G., Sacco A., Fontana M., Bejtka K., Castellino M., Pirri C.F., Cauda V. (2015). Nanobranched ZnO Structure: p-Type Doping Induces Piezoelectric Voltage Generation and Ferroelectric-Photovoltaic Effect. Adv. Mater..

[15] Tu Z., Yang G., Song H., Wang C. (2017). Amorphous ZnO quantum dot/mesoporous carbon bubble composites for a high-performance lithium-ion battery anode. ACS Appl. Mater. Interfaces.

[16] Zhang P., Wu J., Zhang T., Wang Y., Liu D., Chen H., Ji L., Liu C., Ahmad W., Chen Z.D. (2018). Perovskite Solar Cells with ZnO Electron-Transporting Materials. Adv. Mater..

[17] Ahmed T., Edvinsson T. (2020). Optical Quantum Confinement in Ultrasmall ZnO and the Effect of Size on Their Photocatalytic Activity. J. Phys. Chem. C.

[18] Sahoo D., Mandal A., Mitra T., Chakraborty K., Bardhan M., Dasgupta A.K. (2018). Nanosensing of Pesticides by Zinc Oxide Quantum Dot: An Optical and Electrochemical Approach for the Detection of Pesticides in Water. J. Agric. Food Chem..

[19] Lee G., Seo Y.D., Jang J. (2017). ZnO quantum dot-decorated carbon nanofibers derived from electrospun ZIF-8/PVA nanofibers for high-performance energy storage electrodes. Chem. Commun..

[20] Li L., Gu L., Lou Z., Fan Z., Shen G. (2017). ZnO quantum dot decorated Zn2SnO4 nanowire heterojunction photodetectors with drastic performance enhancement and flexible ultraviolet image sensors. ACS Nano.

[21] Gong M., Liu Q., Cook B., Kattel B., Wang T., Chan W.L., Ewing D., Casper M., Stramel A., Wu J.Z. (2017). All-printable ZnO quantum dots/graphenevan der Waals heterostructures for ultrasensitive detection of ultraviolet light. ACS Nano.

[22] Cai X., Luo Y., Zhang W., Du D., Lin Y. (2016). pH-sensitive ZnO quantum dots-doxorubicin nanoparticles for lung cancer targeted drug delivery. ACS Appl. Mater. Interfaces.

[23] Daumann S., Andrzejewskii D., Marcantonio M.D., Hagemann U., Wepfer S., Vollkommer F., Bacher G., Epple E., Nannen E. (2017). Water-free synthesis of ZnO quantum dots for application as an electron injection layer in light-emitting electrochemical cells. J. Mater. Chem. C.

[24] Liu J., Wang Y., Shen J., Liu H., Li J., Wang A., Hui A., Munir H.A. (2020). Superoxide anion: Critical source of high performance antibacterial activity in Co-Doped ZnO QDs. Ceram. Int..

[25] Lee J.H., Baek S.E., Lee H.S., Khang D.Y., Lee W. (2021). Soft-lithographically line-patterned In-doped ZnO quantum dots with hydrothermally grown ZnO nanocolumns for acetone detection. Sens. Actuators B Chem..

[26] Montes-de-Oca L.M., Hernandez-Prudencio A., Borjas-García S.E., Espinosa G., Gomez-Ortiz N.M., Martinez-Torres P. (2018). Controlled Synthesis and Surface Functionalization of ZnO Quantum Dots. Microsc. Microanal..

[27] Ouyang K., Xu B., Yang C., Wang H., Zhan P., Xie S. (2022). Synthesis of a novel Z-scheme Ag/WO_3_/g-C_3_N_4_ nanophotocatalyst for degradation of oxytetracycline hydrochloride under visible light. Mater. Sci. Semicond. Process..

[28] Zhong S., Wang Y., Li S., Wang S., Que X., Sheng L., Peng J., Zhao L., Yuan L., Zhai M. (2022). Enhanced photo-reduction of chromium (VI) from aqueous solution by nanosheet hybrids of covalent organic framework and graphene-phase carbon nitride. Sep. Purif. Technol..

[29] Wang W., Lv B., Tao F. (2022). NiO/g-C_3_N_4_ composite for enhanced photocatalytic properties in the wastewater treatment. Environ. Sci. Pollut. Res..

[30] Li X., Huang G., Chen X., Huang J., Li M., Yin J., Liang Y., Yao Y., Li Y. (2021). A review on graphitic carbon nitride (g-C_3_N_4_) based hybrid membranes for water and wastewater treatment. Sci. Total Environ..

[31] Li C., Wu X., Shan J., Liu J., Huang X. (2021). Preparation, characterization of graphitic carbon nitride photo-catalytic nanocomposites and their application in wastewater remediation: A review. Crystals.

[32] Huang Q., Wang C., Hao D., Wei W., Wang L., Ni B.-J. (2021). Ultralight biodegradable 3D-g-C_3_N_4_ aerogel for advanced oxidation water treatment driven by oxygen delivery channels and triphase interfaces. J. Clean. Prod..

[33] Leelavathi H., Abirami N., Muralidharan R., Kavitha H.P., Tamizharasan S., Arulmozhi R. (2021). Sunlight-assisted degradation of textile pollutants and phytotoxicity evaluation using mesoporous ZnO/gC_3_N_4_ catalyst. RSC Adv..

[34] Gao X., Ai L., Wang L., Ju Y., Liu S., Wang J., Fan H. (2022). The stable and elastic graphitic carbon nitride/polyvinyl alcohol photocatalytic composite sponge: Simple synthesis and application for wastewater treatment. J. Environ. Chem. Eng..

[35] Jirickova A., Jankovsky O., Sofer Z., Sedmidubsky D. (2022). Synthesis and Applications of Graphene Oxide. Materials.

[36] Tiwari S., Purabgola A., Kandasubramanian B. (2020). Functionalised graphene as flexible electrodes for polymer photovoltaics. J. Alloy. Compd..

[37] Sangeetha M., Madhan D. (2020). Ultra sensitive molybdenum disulfide (MoS_2_)/graphene based hybrid sensor for the detection of NO_2_ and formaldehyde gases by fiber optic clad modified method. Opt. Laser Technol..

[38] Zheng J., Li J., Zhang L., Chen X., Huang H. (2020). Post-graphene 2D materials-based antimicrobial agents: Focus on fabrication strategies and biosafety assessments. J. Mater. Sci..

[39] Ming H.D., Feng Y.D., Fei H., Liu J.L. (2018). Electron drift velocity and mobility in graphene. Front. Phys..

[40] Wang J., Tan H., Xiao D., Navik R., Goto M., Zhao Y. (2020). Preparation of waterborne graphene paste with high electrical conductivity. Chem. Phys. Lett..

[41] Ying L., Hui L., Shiwei W., Liu W. (2020). Tuning the optical nonlinearity of graphene. J. Chem. Phys..

[42] Li Y., Li H., Wu S., Liu W. (2021). Chemical Stability of (3,1)-Chiral Graphene Nanoribbons. ACS Nano.

[43] Liang H., Hua P., Zhou Y., Fu J., Tang J., Niu J. (2019). Fabrication of Cu/RGO/MoS_2_ nanohybrid with energetic visible-light response for degradation of rhodamine B. Chin. Chem. Lett..

[44] Lei D., Zhang Q., Liu N., Su T., Wang L., Ren Z., Zhang Z., Su J., Gao Y. (2021). Self-Powered Graphene Oxide Humidity Sensor Based on Potentiometric Humidity Transduction Mechanism. Adv. Funct. Mater..

[45] Zhou Y., Xue C., Gan L., Owens G., Chen Z. (2023). Antibacterial activity of reduced graphene oxide prepared by microbe. Mater. Today Sustain..

[46] Chen Q., Wang Z., Jin H., Zhao X., Feng H., Li P., He D. (2023). Compressed Graphene Assembled Film with Tunable Electrical Conductivity. Materials.

[47] Chen L., Li N., Zhang M., Li P., Lin Z. (2017). Effect of preparation methods on dispersion stability and electrochemical performance of graphene sheets. J. Solid State Chem..

[48] Chen S., Wang W., Hao Y., Meng J., Zhao Y., Wang S., Serge Z., Xu H. (2022). H+/g-C_3_N_4_/GO-COOH Composited by Acid-Treated g-C_3_N_4_ and Functionalized Graphene Oxide for Efficient Photocatalytic H_2_ Production. Energy Fuels.

[49] Jung H., Pham T.T., Shin E.W. (2019). Effect of g-C_3_N_4_ precursors on the morphological structures of g-C_3_N_4_/ZnO composite photocatalysts. J. Alloy. Compd..

[50] Li J., Tang Y., Jin R., Meng Q., Chen Y., Long X., Wang L., Guo H., Zhang S. (2019). Ultrasonic-microwave assisted synthesis of GO/g-C_3_N_4_ composites for efficient photocatalytic H_2_ evolution. Solid State Sci..

[51] Ravichandran K., Uma R., Sriram S., Balamurgan D. (2017). Fabrication of ZnO: Ag/GO composite thin films for enhanced photocatalytic activity. Ceram. Int..

[52] Zhang X., Shao C., Zhang Z., Li J., Zhang P., Zhang M., Mu J., Guo Z., Liang P., Liu Y. (2011). In situ Generation of Well-Dispersed ZnO Quantum Dots on Electrospun Silica Nanotubes with High Photocatalytic Activity. ACS Appl. Mater. Interfaces.

[53] Atchudan R., Edison T., Perumal S., Karthikeyan K., Lee Y. (2016). Facile synthesis of zinc oxide nanoparticles decorated graphene oxide composite via simple solvothermal route and their photocatalytic activity on methylene blue degradation. J. Photochem. Photobiol. B Biol..

[54] Zhang L., Yin L., Wang C., Lun N., Qi Y. (2010). Sol-gel growth of hexagonal faceted ZnO prism quantum dots with polar surfaces for enhanced photocatalytic activity. ACS Appl. Mater. Interfaces.

[55] Sun L.-W., Shi H.-Q., Li W.-N., Xiao H.-M., Fu S.-Y., Cao X.-Z., Li Z.-X. (2012). Lanthanum-doped ZnO quantum dots with greatly enhanced fluorescent quantum yield. J. Mater. Chem..

[56] Zhao L.-H., Zhang R., Zhang J., Sun S.-Q. (2012). Synthesis and characterization of biocompatible ZnO nanoparticles. Cryst. Eng. Comm..

[57] Vinogradov A.V., Vinogradov V.V. (2014). Low-temperature sol–gel synthesis of crystalline materials. RSC Adv..

[58] Sowik J., Miodyńska M., Bajorowicz B., Mikołajczyk A., Lisowski W., Klimczuk T., Kaczor D., Medynska A.Z., Malankowska A. (2018). Optical and photocatalytic properties of rare earth metal-modified ZnO quantum dots. Appl. Surf. Sci..

[59] Chennakesavulu K., Madhusudhana M., Reddy G., Rabel A.M., Brijitta J., Vinita V., Sasipraba T., Sreeramulu J. (2015). Synthesis, characterization and photo catalytic studies of the composites by tantalum oxide and zinc oxide nanorods. J. Mol. Struct..

[60] Ghaderi A., Abbasi S., Farahbod F. (2015). Synthesis of SnO_2_ and ZnO nanoparticles and SnO_2_-ZnO hybrid for the photocatalytic oxidation of methyl orange. Iran. J. Chem. Eng. (IJChE).

[61] Chen L., Li J., Yuan L., Huang C., Tran T.T., Cai Q. (2013). Synthesis and photocatalytic application of Au/Ag nanoparticle-sensitized ZnO films. Appl. Surf. Sci..

[62] Ali M.M., Chauveau J., Bretagnon T. (2018). Evidence of exciton complexes in non polar ZnO/(Zn,Mg)O A-plane quantum well. Superlattice. Microst..

[63] Dong F., Zhao Z., Xiong T., Ni Z., Zhang W., Sun Y., Ho W.-K. (2013). In situ construction of g-C_3_N_4_/g-C_3_N_4_ metal-free heterojunction for enhanced visible-light photocatalysis. ACS Appl. Mater. Interfaces.

[64] Ren C., Yang B., Wu M., Xu J., Fu Z., Yan L., Guo T., Zhao Y., Zhu C. (2010). Synthesis of Ag/ZnO nanorods array with enhanced photocatalytic performance. J. Hazard. Mater..

[65] Niu F.X., Wang Y.X., Zhang Y.T., Xie S.K., Ma L.R., Mao Y.P. (2019). A hierarchical architecting of PANI/APTES/SiC nano-composites with tunable dielectric for lightweight and strong microwave absorption. J. Mater. Sci..

[66] Hijazi M., Stambouli V., Rieu M., Barnier V., Tournier G., Demes T., Viricelle J.P., Pijolat C. (2018). Synthesis and characterization of tin dioxide thick film modiled by APTES in vapor and liquid phases. J. Mater. Sci..

[67] Mousavi-Kamazani M. (2019). Facile sonochemical-assisted synthesis of Cu/ZnO/Al_2_O_3_ nanocomposites under vacuum: Optical and photocatalytic studies. Ultrason. Sonochem..

[68] Dong F., Wu L., Sun Y., Fu M., Wu Z., Lee S. (2011). Efficient synthesis of polymeric g-C_3_N_4_ layered materials as novel efficient visible light driven photocatalysts. J. Mater. Chem..

[69] Wang H., Liu X., Han S. (2016). The synthesis of Ag–ZnO nanohybrid with plasmonic photocatalytic activity under visible light irradiation: The relationship between tunable optical absorption, defect chemistry and photocatalytic activity. Cryst. Eng. Commun..

[70] Duan J., Chen S., Jaroniec M., Qiao S. (2015). Porous C_3_N_4_ Nanolayers@N-graphene flms as catalyst electrodes for highly efficient hydrogen evolution. ACS Nano.

[71] Vinayagam R., Selvaraj R., Arivalagan P., Varadavenkatesan T. (2020). Synthesis, characterization and photocatalytic dye degradation capability of Calliandra haematocephala -mediated zinc oxide nanoflowers. J. Photochem. Photobiol. B Biol..

[72] Abbas A., Zuhra M., Aijaz B. (2023). Efficient photocatalytic degradation of industrial wastewater dye by *Grewia asiatica* mediated zinc oxide nanoparticles. Optik.

[73] Nomita K.D., Anju S.D., Joychandra W.S., Jugeshwar K.S. (2021). Nickel doped zinc oxide with improved photocatalytic activity for Malachite Green Dye degradation and parameters affecting the degradation. J. Mater. Sci. Mater. Electron..

[74] Abebe B., Prakash O.Y., Tania D. (2016). Photocatalytic degradation of methylene blue dye by zinc oxide nanoparticles obtained from precipitation and sol-gel methods. Environ. Sci. Pollut. Res. Int..

[75] Yusuff S.A., Bello A.K., Azeez M.T. (2020). Photocatalytic degradation of an anionic dye in aqueous solution by visible light responsive zinc oxide-termite hill composite. React. Kinet. Mech. Catal..

[76] Srishti K., Kiran M., Sudhish K., Rakshit A., Chetna A. (2019). Kinetics of sonophotocatalytic degradation of an anionic dye nigrosine with doped and undoped zinc oxide. Water Sci. Technol. J. Int. Assoc. Water Pollut. Res..

[77] Kumar R.V., Vinoth S., Baskar V., Arun M., Gurusaravanan P. (2022). Synthesis of zinc oxide nanoparticles mediated by *Dictyota dichotoma* endophytic fungi and its photocatalytic degradation of fast green dye and antibacterial applications. S. Afr. J. Bot..

[78] Inderyas A., Bhatti I.A., Ashar A., Ashraf M., Ghani A., Yousaf M., Mohsin M., Ahmad M., Rafique S., Masood N. (2020). Synthesis of immobilized ZnO over polyurethane and photocatalytic activity evaluation for the degradation of azo dye under UV and solar light irardiation. Mater. Res. Express.

[79] Nida A., Shehzadi S., Samreen F., Tasneem F. (2023). Photocatalytic Degradation of Synthetic Dyes Using Cyanobacteria-Derived Zinc Oxide Nanoparticles. BioNanoScience.

[80] Singh J., Kaur S., Kaur G., Basu S., Rawat M. (2019). Biogenic ZnO nanoparticles: A study of blueshift of optical band gap and photocatalytic degradation of reactive yellow 186 dye under direct sunlight. Green Process. Synth..

